# The difference in quasi-induced exposure to crashes involving various hazardous driving actions

**DOI:** 10.1371/journal.pone.0279387

**Published:** 2023-02-02

**Authors:** Guopeng Zhang, Ying Cai, Lei Li

**Affiliations:** 1 College of Engineering, Zhejiang Normal University, Jinhua, Zhejiang, China; 2 Key Laboratory of Urban Rail Transit Intelligent Operation and Maintenance Technology & Equipment of Zhejiang Province, Zhejiang Normal University, Jinhua, Zhejiang, China; Shahrood University of Technology, ISLAMIC REPUBLIC OF IRAN

## Abstract

In quasi-induced exposure (QIE) theory, the presence of hazardous driving action is the typical determinant of the driver’s responsibility for a crash. However, there is a lack of effort available to analyze the impacts of hazardous actions on the QIE estimate, which may result in estimation bias. Thus, the study aims to explore the difference in QIE to crashes involving various hazardous driving actions. Chi-square test is conducted to examine the consistency of non-responsible party distributions among the crashes involving various hazardous actions. Multinomial logit model and nested logit model are employed to identify the disparities of contributing factors to the actions. Results indicate that: 1) the estimated exposures appear to be inconsistent among the crashes with different hazardous actions, 2) driving cohorts have differential propensities of performing various hazardous actions, and 3) factors such as driver-vehicle characteristics, time, area, and environmental condition significantly affect the occurrence of hazardous actions while the directions and magnitude of the effects show great disparities for various actions. It can be concluded that the QIE estimates are significantly different for crashes involving various hazardous actions, which serves to highlight the importance of clarifying the specific hazardous actions for responsibility assignment in QIE theory.

## Introduction

Crash exposure is an essential measure in traffic safety analysis that indicates the degree of opportunity for a crash to occur. The exposure family mainly includes direct exposure (e.g., vehicle miles traveled (VMT) and average annual daily traffic (AADT)) and indirect exposure. Quasi-induced exposure (QIE) is one of the indirect exposures that has attracted surging popularity among traffic safety researchers in recent decades. Compared to the traditional direct exposures, the QIE poses a great advantage in indicating the crash opportunity of particular driving cohorts at disaggregated levels such as specific periods and locations. Moreover, the exposure estimation in the QIE framework only relies on a crash database without the need to collect extra data such as traffic volume and traveling distance.

Due to the advantages, the QIE method is popularly applied in traffic safety research, particularly in identifying the crash risks of particular driving cohorts such as young drivers, elderly drivers, and commercial vehicles [1–4]. In the QIE theory, the crash risk of the specific driving cohort is presented by the relative crash involvement ratio, which is calculated by the ratio of the proportion of the cohort in the at-fault population to that in the not-at-fault population. In the meanwhile, several studies employed the QIE method to analyze the specific crash types [5, 6]. For instance, Jiang et al. (2021) used the QIE method to interpret the disparity in the injury severities between hit-and-run and non-hit-and-run crashes and found that the underlying exposures are different for two crash types [5]. In addition, the QIE method can be adopted to evaluate the safety effects of traffic measures such as automatic emergency braking systems [7], signal coordination [8], and driver licensing programs [9, 10].

In the application of QIE theory, all crash-involved drivers should be classified into two categories including responsible and non-responsible drivers. Then, the crash exposure can be estimated according to the distribution of the no-responsible driving parties which are assumed to be representative of the overall driving population (i.e., the so-called “not-at-fault assumption”). There has been a variety of studies [11–16] devoted to validating the not-at-fault assumption of QIE theory. The relevant methods mainly include 1) comparing the QIE with direct exposure (e.g., VMT) [11], 2) comparing the distributions of non-responsible parties among crashes with different responsible cohorts [17], and 3) comparing the distributions of non-responsible drivers between two- and three-or-more-vehicle crashes [12–15]. A large body of studies have verified the “not-at-fault assumption” with the use of statistical testing methods such as coefficients of variation [17], Chi-square [11, 12], Wilcoxon Mann-Whitney [13], and Z tests [15]. In contrast, several studies [16, 18] reported that the underlying assumption might not hold under several scenarios, e.g., specific injury severity levels, functional roadway classification, and eco-regions. It needs to mention that the basic premise of the exposure validation is that the crash responsibility has been accurately assigned to each crash-involved driver. The different responsibility assignment methods can consequentially result in a discrepancy in the validation results of the QIE assumption [19].

Typically, there are two kinds of evidence commonly adopted for the responsibility assignment, i.e., the issuance of police citation and the presence of hazardous driving action before the vehicular collision. For the former, a driver would be treated as the responsible party if he/she received a police citation. Here, the citation is determined according to the police officer’s judgment on the traffic violation of the driver. Several previous studies [19–21] pointed out the potential issues of using police citations to allocate crash responsibility. For instance, the issuance of police citations may be subject to “negative halo effects” [21] that specific cohorts (e.g., with suspended/revoked driving licenses, drug usage, and alcohol involvement) have relatively higher probabilities of receiving citations even though they do not cause the crashes. Moreover, it is found that the issuance of police citations can be affected by several factors such as driver gender, age, and injury severity [16, 19]. Particularly, the police officers would not issue citations to the deceased drivers in most cases [18]. The aforementioned issues can consequently bring about the bias of exposure estimation; thus, the police citation-based assignment confronts great challenges in the application of the QIE technique. In this regard, Zhang et al. [22] developed ensemble machine learning that considers both police citation and relevant factors to improve the accuracy of citation-based responsibility assignments.

Another responsibility determination method is based on hazardous driving actions performed by drivers. It is more recommended by safety researchers since the QIE is a behavior-oriented technique [16, 22, 23] and hazardous driving action is the predominant factor affecting traffic crashes [24–26]. Under this assignment method, a driver would be treated as the responsible party if he/she performs any hazardous driving action that directly leads to the vehicle collision. Notwithstanding, the behavior-based method also faces potential concerns in its application. There are many types of hazardous driving actions related to the risk of collision, such as speeding, running red lights, failing to yield, and improper turning. However, these hazardous actions are difficult to be fully taken into account during the data collection. According to the statistics of the Michigan crash data [27], about 4.2% of the information on the driver’s hazardous actions is missing. Meanwhile, 5.2% of the hazardous actions are classified into the category of “others” because they could not be determined. Compared to the police citation, the hazardous action for the responsibility assignment is more difficult to be clearly defined [22]. The missing or unclear information on the hazardous actions may affect the consequential validation of the not-at-fault assumption and QIE estimate. To the best knowledge, there are few studies available to analyze the impacts of hazardous action selection on the QIE estimates. The responsibility assignment based on different hazardous actions may result in disparities in exposure estimates, which deserves to be paid more attention.

In summary, the comprehensive review has revealed that the majority of the existing studies are devoted to validating the “not-at-fault assumption” of QIE theory, in which hazardous driving actions are typically adopted as the determinant to assign crash responsibility to drivers. The responsibility assignment is a key step that can considerably affect the assumption validation and the exposure estimation. However, there are few studies available to discuss which driving actions should be taken into account for responsibility assignment. To fill this gap, the study aims to explore the difference in QIE estimates for crashes with various hazardous actions. The Chi-square test would be employed to examine the consistency of the crash exposures, in which the null hypothesis is that the exposures are consistent for crashes involving different hazardous actions. Then, statistical regression models would be adopted to explore the factors contributing to the occurrence of hazardous driving actions. The findings can serve to clarify the specific hazardous actions for responsibility assignment in QIE theory.

## Methodology

### Quasi-induced exposure theory

QIE technique has been frequently employed to estimate the crash exposure of specific driving cohorts. In the QIE theory, there are two underlying assumptions: 1) the responsibility for a crash needs to be clearly assigned to each driver. Thus, all the crash-involved drivers are classified into two categories, i.e., the responsible drivers (D1) and the non-responsible drivers (D2). Typically, the QIE theory is applied to two-vehicle crashes with a D1 driver and a D2 driver, and 2) the D2s in the crashes are assumed to be the random selection of the overall driving population. Thus, D2 is the target exposure to be estimated.

According to the previous QIE studies [8, 28], the crash propensity of a specific driving cohort is presented by the relative crash involvement ratio (IR).

IRi=D1i∑iD1iD2i∑iD2i (1)

where *IR*_*i*_ is the crash propensity of specific driving cohort *i*, *D*1_*i*_ and *D*2_*i*_ denote the number of D1 and D2 drivers for cohort *i*, respectively, and ∑_*i*_ *D*1_*i*_ and ∑_*i*_ *D*2_*i*_ denote the total number of D1 and D2 drivers in the two-vehicle crashes, respectively.

### Chi-square test

The study attempts to employ the Chi-square test to examine the consistency of the exposures to crashes with different hazardous actions. The Chi-square test has been developed and popularly used in previous QIE studies [13, 16]. In the framework of the Chi-square test, the null hypothesis is that observations for comparison have the same distribution. Thus, the study assumes that the estimated exposures (D2 distribution) are consistent for crashes involving different hazardous actions. The Chi-square *χ*^2^ can be calculated by:

$$\chi^{2}=\sum_{i=1}^{r}\sum_{j=1}^{c}\frac{{\left(f_{ij}-e_{ij}\right)}^{2}}{e_{ij}}$$ (2)

where *r* is the total number of driver-vehicle categories, *c* is the total number of hazardous action types, *f*_*ij*_ is the number of D2 for driving cohort *i* in the scenario of the *j*^th^ hazardous action, and *e*_*ij*_ is the expected number of D2 for driving cohort *i* in the scenario of the *j*^th^ hazardous action, which is defined as:

$$e_{ij}=f_{i}∙f_{j}/n$$ (3)

Where *f*_*i*_ is the *i*^th^ marginal row frequency, *f*_*j*_ is the *j*^th^ marginal column frequency, and *n* is the total number of D2s. In the field of statistics, *p* value <0.05 is typically used to identify the significant difference between comparative data. Particularly, a large number of previous QIE studies adopted this criterion to reject the null hypothesis of the Chi-square test [5, 11, 16, 18]. Thus, the study uses *p* value <0.05 as evidence to reject the null hypothesis.

### Statistical models

Since whether a driver would involve in a specific hazardous action is a discrete choice, two discrete choice models, i.e., the multinomial logit model and nested logit model, are used to reveal the differential factors contributing to the occurrence of various hazardous actions.

#### Multinomial logit model

The multinomial logit model is a typical discrete-choice model that is frequently employed in previous traffic safety research [29–31]. In the multinomial logit model, there is a hypothesis that the probability ratio for the two alternatives depends only on their characteristics and not on those of others, which is called the IIA hypothesis. In this study, we suppose that there are *J* different hazardous driving actions that drivers may perform. The individual driver is supposed to perform the action with the highest level of utility. The utility of alternative action *j* is presented by

$$U_{j}=\beta_{j}^{⊺}x_{j}+\varepsilon_{j}=V_{j}+\varepsilon_{j}$$ (4)

where $\beta_{j}^{⊺}$ is the coefficient of the variable *x*_*j*_ and *ε*_*j*_ is the error term that includes the impacts of all the unobserved variables, which follows a Gumbel distribution. Then, the probability of choosing alternative *j* can be calculated by

$$P(j)=\frac{e^{j}}{\sum_{k}e^{V_{k}}}$$ (5)

#### Model

It is worth mentioning that the IIA hypothesis of the multinomial logit model does not always hold in fact. Compared to the standard multinomial logit model, the nested logit model is more flexible for relaxing the IIA assumption. It is a generalization of the multinomial logit model that is based on the idea that some alternatives (hazardous actions) can be joined in several specific groups (called nests) that share unobserved impacts. The nested logit model has also been developed in previous studies on traffic safety [32–34]. We suppose that hazardous actions can be classified into *M* different nests. Then, the probability of choosing hazardous action *j* can be computed by

P(j)=P(j|l)×P(l) (6)

where the first term *P*(*j*|*l*) is the conditional probability of choosing hazardous action *j* if the nest *l* is chosen. The second term *P*(*l*) is the marginal probability of choosing the nest *l*.

$$P(j|l)=\frac{e^{Z_{j}/\lambda_{l}}}{\sum_{k\in B_{l}}e^{Z_{k}/\lambda_{l}}}$$ (7)

$$P(l)=\frac{e^{W_{l}+\lambda_{l}I_{l}}}{\sum_{m=1}^{M}e^{W_{m}+\lambda_{m}I_{m}}}$$ (8)

where *Z*_*j*_ and *W*_*l*_ are the parts of the utility being specific to the hazardous action *j* and the nest *l*, respectively; *B*_*l*_ is the set of hazardous actions belonging to the nest *l*; *λ*_*l*_ indicates the correlation of hazardous action in the nest *l*, which is also known as the IV parameter falling between 0 and 1. A higher value of IV means greater independence and less correlation; and $I_{l}=ln\sum_{k\in B_{l}}e^{Z_{k}/\lambda_{l}}$, which is called the inclusive value.

**Fig 1:**
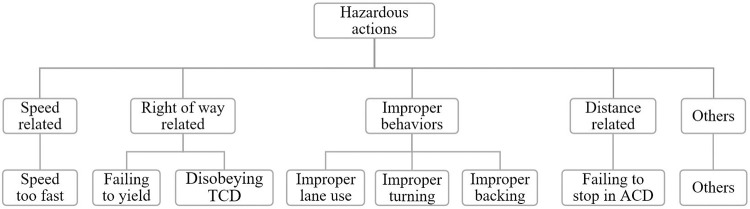
Structure of the nested logit model.

Fig 1 illustrates the structure of the nested logit model in the study. All hazardous actions are classified into five categories, including speed-related, right-of-way-related, improper, distance-related, and other actions. Here, the right-of-way-related actions include failing to yield and disobeying traffic control devices (TCD). The improper actions can be further divided into improper lane use, turning, and backing. The distance-related hazardous action means failing to stop in assured clear distance (ACD).

#### Goodness of fit

Akaike Information Criterion (AIC) is employed to reflect the goodness of fit of regression models [35–37].

AIC=−2*ln(L)+2k (9)

where *L* is the value of the likelihood and *k* is the number of regression parameters.

### Data

The QIE estimation of particular driving cohorts is based on traffic crash data. The study extracts the crashes from the Michigan crash database (2014) which is provided by the Michigan Department of Transportation. Each traffic crash is investigated and recorded in the crash database in standard procedures. The crash-related variables have been clearly defined and coded in the crash database. According to the statistics, there are nearly 300,000 traffic crashes occurred in Michigan State each year. The large sample size of crashes is sufficient to ensure the stability of the exposure estimate. Moreover, the database contains detailed information on each driving party involved in the crashes, such as the driver’s age, gender, hazardous driving action, and vehicle type. The information on the hazardous action can be used as evidence for crash responsibility assignment in QIE theory. In addition, the crash database also includes extra information such as the time, location, and environmental condition, which can serve to identify the factors influencing the propensity of specific hazardous actions.

According to the requirement of QIE theory, it is desirable to conduct data cleaning procedures. The criteria are referred to previous QIE studies [16, 22], as follows.

1. it extracts two-vehicle crashes with a responsible driver and a non-responsible driver. Here, the responsibility of a driver is determined according to the presence of hazardous action;
2. the crashes involving the missing variables of three key driver-vehicle characteristics (i.e., driver age, gender, and vehicle type) are removed from the database; and
3. the study focused on two-vehicle crashes, thus, the crashes involving motorcycles, bikes, and animals are not included in the analysis.

After the data cleaning procedures, there are 114,666 crash samples finally remained for the data analysis. The Michigan crash database recorded many types of hazardous actions involved in the crashes. The study only focuses on the actions that are frequently observed including speed too fast, failing to yield, disobeying TCD, improper lane use, improper turning, improper backing, and failing to stop in ACD. The actions that are not clearly determined or have few samples are classified into the category of “other actions.” As for the variable selection, the study considers three key driver-vehicle variables (e.g., driver age, gender, and vehicle type) to analyze the exposures of specific driving cohorts. In addition, the road and environmental conditions are also taken into account to identify the contributory factors affecting the occurrence of hazardous driving actions. Table 1 presents the definitions of the variables in the modeling exercises.

**Table 1:** Definitions of variables for the Michigan crash database.

Variables	Definition
**Hazardous actions**	The hazardous action associated with the at-fault driver (1 if speed too fast (H1), 2 if failing to yield (H2), 3 if disobeying TCD (H3), 4 if improper lane use (H4), 5 if improper turning (H5), 6 if improper backing (H6), 7 if failing to stop in ACD (H7), 8 if other actions (H8))
**D1Age**	The age of the responsible driver (1 if 15–30, 2 if 31–60, 3 if >60)
**D1Gender**	The gender of the responsible driver (1 if male, 0 if female)
**D1Type**	The vehicle type of the responsible party (1 if passenger car & van, 2 if pick up, 3 if heavy vehicle)
**D2Age**	The age of the non-responsible driver (1 if 15–30, 2 if 31–60, 3 if >60)
**D2Gender**	The gender of the non-responsible driver (1 if male, 0 if female)
**D2Type**	The vehicle type of the non-responsible party (1 if passenger car & van, 2 if pick up, 3 if heavy vehicle)
**Speed limit**	The speed limit on the roadway whether the crash occurs (1 if <35 mph, 2 if 35–50 mph, 3 if >50 mph)
**Peak hour**	Whether the crash occurs between 7–8 am or 5–7 pm (1 if yes, 0 if no)
**Weekday**	Whether the crash occurs on a weekday (1 if yes, 0 if no)
**Intersection**	Whether the crash occurs at an intersection (1 if yes, 0 if no)
**Area**	Whether the crash occurs in the urban area (1 if yes, 0 if no)
**Light**	The lighting condition when the crash occurs (1 if daylight, 0 if others)
**Weather**	The weather condition when the crash occurs (1 if clear, 2 if cloudy, 3 if rainy & snowy)

## Results

### Comparison of D2 distribution

**Fig 2:**
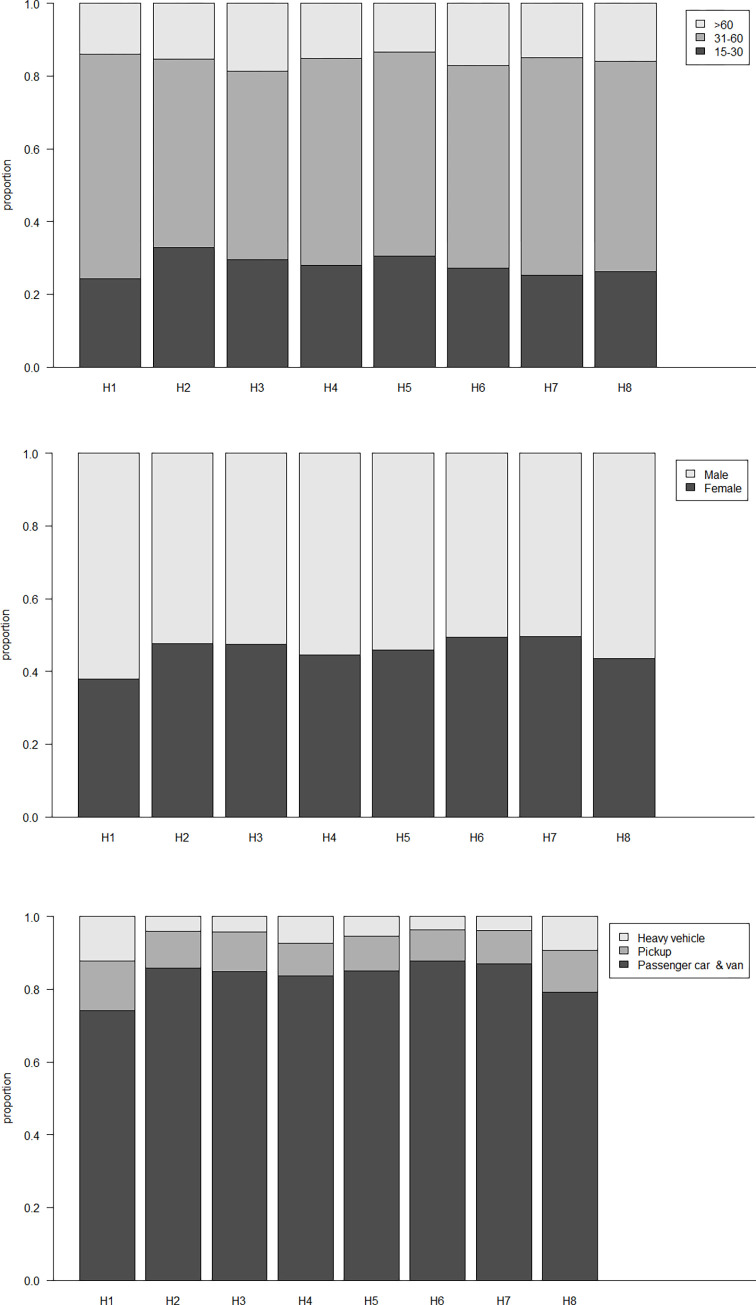
D2 distribution disaggregated by hazardous action of D1. (a) driver age, (b) driver gender, (c) vehicle type.

Fig 2 presents the D2 distribution in terms of three driver-vehicle characteristics disaggregated by the hazardous action of D1. The information displayed indicates that the D2 distributions appear to be different under various scenarios. In the aspect of driver age, young driver accounts for the larger proportion (32.9%) in crashes involving failing to yield as opposed to those in other scenarios. Elderly drivers have a larger proportion (18.7%) in crashes involving disobeying traffic control devices compared to those involving other hazardous actions. With respect to driver gender, male drivers typically have larger crash exposure than female drivers. Particularly in the scenario of speeding-involved crashes, the proportion of male drivers is more than 60%, which is obviously higher than those in other scenarios. As for the vehicle type, the proportion of passenger cars & vans is typically larger than those of pickups and heavy vehicles in all the scenarios since the majority of vehicles driven on the roads is passenger car & van in reality. Notwithstanding, it is observed that pickups and heavy vehicles have large exposures to be collided with a speeding vehicle as opposed to vehicles involving other hazardous actions. It can be explained by the greater difference in traveling velocity between the crash-involved vehicles that are more likely to incur traffic crashes [38]. The disparity in D2 distribution implies that quasi-induced exposures are not consistent for crashes involving various hazardous actions.

Table 2 presents the Chi-square tests of the D2 distribution in terms of three key characteristics, i.e., driver age, gender, and vehicle type. The *p* values of Chi-square tests are consistently less than 0.05, which verifies the significance of disparity in D2 distribution among the crashes with different hazardous actions.

**Table 2:** Chi-square tests of the D2 distribution.

Driver-vehicle groups	H1	H2	H3	H4	H5	H6	H7	H8	Chi-square test
**Age**									
**15–30**	1,857	10,677	1,832	1,761	944	924	11,849	2,299	*χ*^2^ = 814.91
**31–60**	4,701	16,793	3,197	3,597	1,732	1,900	27,982	5,059	*p*<0.001
**>60**	1,074	4,998	1,156	960	417	584	6,977	1,396	
**Gender**									
**Female**	2,896	15,459	2,940	2,809	1,419	1,684	23,187	3,820	*χ*^2^ = 439.42
**Male**	4,736	17,009	3,245	3,509	1,674	1,724	23,621	4,934	*p*<0.001
**Vehicle type**									
**Passenger car & van**	5,664	27,863	5,252	5,284	2,629	2,991	40,675	6,928	*χ*^2^ = 1,682.9
**Pickup**	1,029	3,284	670	564	298	293	4,312	1,013	*p*<0.001
**Heavy vehicle**	939	1,321	263	470	166	124	1,821	813	

To further verify the results, Chi-square tests are also conducted for the D2 distribution with the use of Michigan crash data (2012 and 2013). The results (shown in Tables A1 and A2 in S1 Appendix) indicate that *p* values are consistently less than 0.05 for driver age, gender, and vehicle type, which are in good agreement with the findings from Table 2. Thus, it suffices to demonstrate that the quasi-induced exposures are significantly different for crashes involving various hazardous actions.

### Relative crash involvement ratio

Table 3 summarizes the IR values of various driving cohorts disaggregated by hazardous action. The information displaced indicates that young drivers usually have a higher propensity to cause traffic crashes, justified by the relatively higher IR values (>1). It may be attributed to the higher propensities of aggressive driving behaviors [9] and risk-taking attitudes [17]. Especially for the hazardous action of speeding, the IR value of young drivers is up to 2.109. In contrast, the mid-age drivers have relatively lower crash risks for all the scenarios since IR values are consistently less than 1. The reason is that mid-age drivers usually have rich driving experiences and good physical conditions to avoid the risks of traffic crashes. As for the elderly drivers, the IR values are not stable across the scenarios. To be specific, elderly drivers are more likely to perform the actions of failing to yield, improper lane use, turning, and backing while they have less propensity of speeding, disobeying traffic control devices, failing to stop in ACD, and other actions. The phenomenon is possibly due to the poor physical condition and good driving attitude of elderly drivers. In terms of driver gender, female drivers have more likelihood of speeding while male drivers are inclined to involve improper backing and failing to stop in ACD. As for vehicle type, passenger car & van is more likely to be engaged in speeding as opposed to pickup and heavy vehicle. The pickup has a higher propensity for improper backing, failing to stop in ACD, and other hazardous actions. Heavy vehicles have a considerably high propensity for improper turning and backing. Especially for improper backing, the IR value of heavy vehicles is extremely high (3.911). It is possibly due to the large size and poor mobility that can interrupt traffic and lead to vehicle collisions [39, 40]. The differential IR values suggest that the specific driving cohorts have different propensities for performing various dangerous driving actions and causing crashes.

**Table 3:** IRs of driving cohorts.

Driver-vehicle groups	H1	H2	H3	H4	H5	H6	H7	H8
**Age**								
**15–30**	2.109	1.217	1.436	1.239	1.162	1.065	1.836	1.428
**31–60**	0.666	0.744	0.770	0.793	0.778	0.919	0.721	0.817
**>60**	0.546	1.397	0.944	1.336	1.556	1.161	0.699	0.959
**Gender**								
**Female**	1.112	1.027	0.996	0.991	0.986	0.679	0.897	0.918
**Male**	0.932	0.975	1.003	1.007	1.012	1.313	1.101	1.063
**Vehicle type**								
**Passenger car & van**	1.143	1.013	1.030	0.999	0.943	0.691	0.995	0.989
**Pickup**	0.889	0.973	0.899	1.004	0.990	2.922	1.152	1.186
**Heavy vehicle**	0.258	0.800	0.654	1.009	1.922	3.911	0.751	0.866

### Regression of statistical models

Tables 4 and 5 present the regression results of the multinomial logit and nested logit models, respectively. Note that the variables of D2Age and D2Gender have been excluded from the models because they are not significant contributors to the occurrence of hazardous actions under most scenarios. For the remained variables, the estimates of the parameters appear to be similar between the two models, which can somewhat serve to verify the validity of the findings.

**Table 4:** The result of multinomial logit model regression.

Variables	H1		H2		H3		H4		H5		H6		H7	
	estimate	*p*	estimate	*p*	estimate	*p*	estimate	*p*	estimate	*p*	estimate	*p*	estimate	*p*
***α***	-1.20	<0.01	1.11	<0.01	-0.92	<0.01	-0.98	<0.01	-1.28	<0.01	-1.23	<0.01	0.38	<0.01
**D1Age (base: 31–60)**														
**15–30**	0.39	<0.01	0.22	<0.01	0.21	<0.01	-0.05	0.20	0.03	0.52	-0.21	<0.01	0.26	<0.01
**>60**	-0.53	<0.01	0.47	<0.01	0.23	<0.01	0.29	<0.01	0.38	<0.01	0.23	<0.01	-0.36	<0.01
**D1Gender**	-0.06	0.08	-0.23	<0.01	-0.15	<0.01	-0.09	0.01	-0.15	<0.01	0.12	0.01	-0.05	0.03
**D1Type (base: passenger car & van)**														
**Pickup**	-0.28	<0.01	-0.28	<0.01	-0.30	<0.01	-0.38	<0.01	-0.23	<0.01	0.81	<0.01	-0.23	<0.01
**Heavy vehicle**	-1.09	<0.01	-0.75	<0.01	-0.91	<0.01	-0.04	0.55	0.45	<0.01	0.86	<0.01	-0.95	<0.01
**D2Type (base: passenger car & van)**														
**Pickup**	0.13	0.01	-0.16	<0.01	-0.06	0.31	-0.24	<0.01	-0.17	0.02	-0.42	<0.01	-0.26	<0.01
**Heavy vehicle**	0.22	<0.01	-0.72	<0.01	-0.57	<0.01	-0.22	<0.01	-0.52	<0.01	-1.17	<0.01	-0.81	<0.01
**Speed limit (base: 35–50 mph)**														
**<35 mph**	-0.13	0.01	-0.39	<0.01	-0.21	<0.01	-0.31	<0.01	-0.02	0.7	0.96	<0.01	-0.62	<0.01
**>50 mph**	0.96	<0.01	-0.55	<0.01	-0.54	<0.01	-0.28	<0.01	-0.97	<0.01	-0.73	<0.01	-0.15	<0.01
**Peak hour**	-0.01	0.87	0.07	0.02	-0.17	<0.01	-0.02	0.52	<0.01	0.98	-0.15	<0.01	0.24	<0.01
**Weekday**	0.09	0.04	0.13	<0.01	-0.12	<0.01	0.17	<0.01	-0.03	0.61	0.07	0.18	0.36	<0.01
**Intersection**	-0.12	<0.01	0.64	<0.01	1.54	<0.01	-0.04	0.24	0.58	<0.01	-0.13	<0.01	0.36	<0.01
**Area**	0.07	0.08	0.07	0.02	0.17	<0.01	0.96	<0.01	0.35	<0.01	-1.11	<0.01	0.84	<0.01
**Light**	0.08	0.03	0.21	<0.01	-0.08	0.03	0.26	<0.01	0.13	0.01	0.46	<0.01	0.46	<0.01
**Weather (base: clear)**														
**Cloudy**	0.34	<0.01	0.05	0.12	-0.12	<0.01	-0.08	0.04	-0.17	<0.01	-0.07	0.14	0.01	0.62
**Rainy & snowy**	1.57	<0.01	-0.22	<0.01	-0.46	<0.01	-0.40	<0.01	-0.38	<0.01	-0.29	<0.01	0.08	0.01

**Table 5:** The result of nested logit model regression.

Variables	H1		H2		H3		H4		H5		H6		H7	
	estimate	*p*	estimate	*p*	estimate	*p*	estimate	*p*	estimate	*p*	estimate	*p*	estimate	*p*
***α***	-1.20	<0.01	1.13	<0.01	-0.63	<0.01	-0.83	<0.01	-1.08	<0.01	-1.07	<0.01	0.38	<0.01
**D1Age (base: 31–60)**														
**15–30**	0.39	<0.01	0.22	<0.01	0.21	<0.01	-0.05	0.17	0.02	0.69	-0.19	<0.01	0.26	<0.01
**>60**	-0.53	<0.01	0.47	<0.01	0.25	<0.01	0.29	<0.01	0.37	<0.01	0.24	<0.01	-0.36	<0.01
**D1Gender**	-0.06	0.08	-0.22	<0.01	-0.16	<0.01	-0.09	0.01	-0.14	<0.01	0.10	0.03	-0.05	0.03
**D1Type (base: passenger car & van)**														
**Pickup**	-0.28	<0.01	-0.28	<0.01	-0.30	<0.01	-0.34	<0.01	-0.20	<0.01	0.74	<0.01	-0.23	<0.01
**Heavy vehicle**	-1.09	<0.01	-0.75	<0.01	-0.89	<0.01	<0.01	0.99	0.43	<0.01	0.81	<0.01	-0.95	<0.01
**D2Type (base: passenger car & van)**														
**Pickup**	0.13	0.01	-0.16	<0.01	-0.07	0.2	-0.25	<0.01	-0.18	0.01	-0.41	<0.01	-0.26	<0.01
**Heavy vehicle**	0.22	<0.01	-0.71	<0.01	-0.59	<0.01	-0.25	<0.01	-0.51	<0.01	-1.10	<0.01	-0.81	<0.01
**Speed limit (base: 35–50 mph)**														
**<35 mph**	-0.13	0.01	-0.38	<0.01	-0.23	<0.01	-0.25	<0.01	<0.01	1.00	0.87	<0.01	-0.62	<0.01
**>50 mph**	0.96	<0.01	-0.54	<0.01	-0.56	<0.01	-0.31	<0.01	-0.91	<0.01	-0.72	<0.01	-0.15	<0.01
**Peak hour**	-0.01	0.87	0.06	0.03	-0.14	<0.01	-0.03	0.48	-0.01	0.88	-0.14	<0.01	0.24	<0.01
**Weekday**	0.09	0.04	0.13	<0.01	-0.10	0.02	0.16	<0.01	-0.01	0.81	0.07	0.16	0.36	<0.01
**Intersection**	-0.12	<0.01	0.65	<0.01	1.43	<0.01	-0.03	0.41	0.51	<0.01	-0.10	0.03	0.36	<0.01
**Area**	0.07	0.09	0.07	0.02	0.16	<0.01	0.86	<0.01	0.32	<0.01	-0.96	<0.01	0.84	<0.01
**Light**	0.08	0.03	0.20	<0.01	-0.05	0.21	0.26	<0.01	0.15	<0.01	0.44	<0.01	0.46	<0.01
**Weather (base: clear)**														
**Cloudy**	0.34	<0.01	0.04	0.15	-0.10	0.01	-0.08	0.03	-0.16	<0.01	-0.07	0.11	0.01	0.61
**Rainy & snowy**	1.57	<0.01	-0.22	<0.01	-0.43	<0.01	-0.40	<0.01	-0.38	<0.01	-0.29	<0.01	0.08	0.01
**IV**	0.87	<0.01												

The results indicate that the influential factors show conspicuous diversities for different hazardous actions. In the aspects of D1 characteristics, it is found that young drivers are more likely to engage in risky behaviors such as speeding, failing to yield, disobeying TCD, and failing to stop in ACD while they have less propensity of performing improper backing. The elderly drivers are identified to have significantly higher propensities of failing to yield, disobeying TCD, improper lane use, improper turning, and improper backing while they have fewer propensities of speeding and failing to stop in ACD. Regarding the driver gender, female drivers are generally prone to be involved with hazardous actions as opposed to male drivers, except the improper backing. As for the vehicle types, pickups and heavy vehicles are inclined to perform improper backing, while passenger cars & vans are more likely to be involved in other hazardous actions. The above findings are generally consistent with the findings derived from the IR values of specific driving cohorts. In addition, it is also found that the D2 vehicle type can be a contributory factor to crashes with specific hazardous actions. Particularly, the speeding vehicle is prone to collide with an innocent heavy vehicle as opposed to a passenger car or van, which can be interpreted by the greater difference in traveling velocity between the speeding vehicles and heavy vehicles.

Except for the driver-vehicle characteristics, the information displaced also suggests that the propensities of various hazardous actions differ substantially for various circumstances. The actions of failing to yield and stop in ACD are more likely to occur during peak hours, while the actions of disobeying TCD and improper backing indicate the opposite. Another temporal variable “Weekday” is also identified to have differential impacts on the various hazardous actions. In terms of the spatial variables, most hazardous actions are prone to take place on the roadway with a moderate limit (35–50 mph), e.g., failing to yield, disobeying TCD, improper lane use, and failing to stop in ACD, while speeding is significantly linked to the roadway with high-speed limit (>50 mph). Actions such as disobeying TCD, improper turning, and failing to stop in ACD are more frequently observed in the intersection area. The urban area is typically associated with the most hazardous action types (except the action of improper backing) compared to the rural area. As for the environmental condition, the dark condition is a factor significantly increasing the probability of disobeying TCD. While other hazardous actions are generally inclined to take place under daylight conditions. Hazardous actions such as speeding and failing to stop in ACD are more likely to be performed when it is rainy & snowy. The finding can be reasonably explained that vehicles required longer stopping distances to avoid collisions on slippery roads [41, 42]. In contrast, actions such as disobeying TCD, improper lane use, turning, and backing are more likely to occur when it is cloudy.

### Model comparison

To validate the reliability of the regression results, several discrete choice models are conducted for the model comparison, including the multinomial probit model, stereotype logit model, and random parameter multinomial logit model. All these models are suitable for the regression with categorical dependent variables, e.g., the different hazardous actions in this study. Note that the random parameter models are the state-of-the-art methods applied in traffic safety research that consider the unobserved heterogeneity of variables [43–45]. In the random parameter models, the coefficients of variables are assumed to be random to take into account the unobserved heterogeneity of variables. Table 6 presents the Log-likelihood and AIC values to test the goodness of fits of the statistical models. Compared to the multinomial probit model and stereotype logit model, the multinomial logit model appears to has a relatively better goodness of fit according to the larger Log-likelihood value and lower AIC value. The performance indexes of the random parameter multinomial logit model are similar to those of the multinomial logit model. Thus, it can be inferred that the goodness of fit cannot be improved by setting random parameters and the unobserved heterogeneity of variables is not significant in this study. Nonetheless, it is found that the goodness of fit can be improved in the nested logit model in which some hazardous actions are joined in specific groups to share unobserved impacts. The results can be reasonably explained by the existence of correlations among the hazardous actions in certain groups.

**Table 6:** Comparison of the statistical models.

Models	AIC	Log-likelihood
**Multinomial logit model**	343,364.4	-171,560
**Nested logit model**	343,363.2	-171,560
**Multinomial probit model**	343,521.5	-171,642
**Stereotype logit model**	355,979.7	-177,961
**Random parameter multinomial logit model**	343,364.4	-171,560

## Discussion

Quasi-induced exposure (QIE) theory requires the clear-cut assignment of crash responsibility for the crash-involved drivers which is typically determined by hazardous driving actions. The study explores the disparities of QIE to crashes involving different hazardous actions and examines the factors contributing to the occurrence of various hazardous actions. The findings can serve to highlight the importance of clarifying the specific hazardous actions for responsibility assignment in QIE theory.

The Chi-square tests demonstrate that the estimated exposures of driver-vehicle cohorts are significantly different for the crashes with various hazardous actions. Cohorts such as male and mid-age drivers, pickups, and heavy vehicles have relatively larger exposure to crashes involving speeding than those involving other hazardous actions. A feasible interpretation is that the specific driving cohorts are expected to occur in the locations where the corresponding hazardous actions are frequently performed. For instance, heavy vehicles usually travel on specific routes such as freeways where speeding behavior is prone to occur while other hazardous actions have fewer opportunities to be performed [46, 47]. This can be verified by the results of statistical regression that the roadway with a high-speed limit (>50 mph) is significantly linked to the higher probability of speeding while it has negative impacts on all the other risky behaviors. In addition, the psychosocial environment [48] and stress reactions [49] may be different for the driving cohorts, which may eventually affect risky driving behaviors and crash exposures. According to the differential exposure to crashes with various hazardous actions, it can be inferred that the QIE estimate would be biased if some hazardous actions are overlooked during the crash investigation and data collection. Thus, the study emphasizes the importance of fully taking into account the various hazardous actions during the data collection.

The results of the IR calculation indicate that the particular driving cohorts have different propensities for performing various hazardous actions. As anticipated, young drivers have relatively higher risks of involving all kinds of hazardous actions (especially speeding) while mid-age drivers indicate the opposite, which is in line with previous QIE studies [8, 50–52]. A feasible interpretation is that young drivers generally have higher propensities of aggressive driving behaviors and risk-taking attitudes [9, 17]. While for elderly drivers, the propensities of different hazardous actions show noticeable diversities, which implies that QIE estimation and crash risk evaluation for elderly drivers might be biased if the hazardous actions were not fully considered. Similar findings are observed for the driving cohorts of specific driver gender and vehicle types. Particularly, the propensities of improper turning and backing are extremely high for heavy vehicles. Considering the discrepancy of IR values, the study suggests that safety analysis of driving cohorts can be disaggregated by hazardous actions, which can help to identify the specific crash risks associated with the driving cohorts.

The regressions of the multinomial logit and nested logit models indicate that the influential factors show great disparities for the various hazardous actions. Particularly for driver-vehicle characteristics of the responsible parties, it is observed that the direction and the magnitude of the impacts are not consistent across the various scenarios, which can somewhat serve to warrant the presence of disparities in the propensity of involving various actions for specific cohorts. The vehicle types of the innocent parties can also be significant contributors to crashes with specific hazardous actions. Generally, passenger cars & vans are more likely to be collided as opposed to pickups and heavy vehicles. An opposing finding is that speeding vehicles are prone to collide the pickups and heavy vehicles. The displayed information also indicates that the occurrences of hazardous actions are affected by a number of factors regarding time, location, and environmental conditions. Actions such as failing to yield, disobeying TCD, and improper turning are usually performed in the intersection area; thus, the intersection area is identified to be the contributory factor to these driving actions in the regression results. In terms of the light condition, all hazardous actions are inclined to occur under daylight except for the action of disobeying TCD. The finding is somewhat inconsistent with a previous QIE study on red light running crashes that the crash risk is higher in the daytime [53]. A possible reason is that the action of disobeying TCD in the study includes not only running red lights but also other violations related to traffic control devices. As for the weather condition, most hazardous actions are inclined to occur when it is clear. In contrast, crashes involving speeding and failing to stop in ACD are prone to occur when it is rainy or snowy. due to the longer stopping distance required to avoid vehicle collisions [41, 42].

The estimated parameters are similar in the statistical models, which serves to further demonstrate the validity of the findings. Notwithstanding, it is found that the nested logit model has a relatively lower AIC value than the multinomial logit model, which suggests that the nested logit model has better goodness of fit. This may be attributable to the different correlations among the various hazardous actions. In the nested logit model, the hazardous actions in the same nests generally have stronger correlations than those in the different nests. For example, both the actions of failing to yield and disobeying TCD are closely related to the right of way and are prone to occur in intersection areas. Nonetheless, considering that there are a variety of hazardous action types, further efforts can be directed to explore the different classifications of hazardous actions for the nests to achieve better goodness of fit. To further understand the characteristics of hazardous actions, it also calls for developing advanced analytic methods to identify the heterogeneity [54] and spatial effects of hazardous actions (e.g., spatial correlation, spatial heterogeneity, and spillover effect) [55]. In addition, the real-time safety evaluation method is encouraged to be explored to diminish the hazardous actions in time [56].

## Conclusion

Hazardous driving actions are the typical determinant for crash responsibility assignment in QIE theory. With the use of Michigan crash data, the study explores the difference in QIE for crashes with various hazardous actions. The analytic results demonstrate that 1) the exposures are inconsistent among the crashes involving different hazardous actions, 2) driving cohorts have differential propensities for performing the various hazardous driving action, and 3) the occurrences of hazardous actions are influenced by many factors such as driver-vehicle characteristics, time, area, and environmental condition. The findings serve to highlight the importance of clarifying the specific hazardous actions for responsibility assignment so as to improve the accuracy of QIE estimation.

The limitation of the study is that it only compares the exposures between crashes with different hazardous actions, while does not assess the accuracy of exposure estimates by comparison with the exposure truth (e.g., VMT and AADT) due to the lack of relevant data. Future work can be directed to further validate the accuracy of the exposure estimation under specific responsibility assignment criteria when the data of exposure truth is available.

## Supporting information

S1 Data Crash data. (CSV)

S1 Appendix Chi-square tests of the D2 distribution (2012 and 2013). (DOCX)

S1 File (PDF)
