# Supplementary material for: The difference in quasi-induced exposure to crashes involving various hazardous driving actions
Source: PLoS One. 2023 Feb 2;18(2):e0279387. doi: 10.1371/journal.pone.0279387 (PMC9894421; doi:10.1371/journal.pone.0279387)
Supplement: S1 Appendix — (DOCX) [file pone.0279387.s002.docx]

**Appendix**

**Table A1 Chi-square tests of the D2 distribution (2012)**

| **Driver-vehicle groups** | **H1** | **H2** | **H3** | **H4** | **H5** | **H6** | **H7** | **H8** | **Chi-square test** |
| --- | --- | --- | --- | --- | --- | --- | --- | --- | --- |
| **Age** |  |  |  |  |  |  |  |  |  |
| **15-30** | 1,067 | 9,478 | 1,832 | 1,581 | 836 | 754 | 10,592 | 1,979 | $\chi^{2}$=594.07  $p$<0.001 |
| **31-60** | 2,516 | 14,910 | 3,093 | 3,208 | 1,487 | 1,541 | 24,313 | 4,327 |  |
| **>60** | 534 | 4,410 | 994 | 776 | 379 | 475 | 5,942 | 1,078 |  |
| **Gender** |  |  |  |  |  |  |  |  |  |
| **Female** | 1,635 | 14,016 | 2,814 | 2,581 | 1,315 | 1,460 | 20,725 | 3,296 | $\chi^{2}$=282.59  $p$<0.001 |
| **Male** | 2,482 | 14,782 | 3,105 | 2,984 | 1,387 | 1,310 | 20,122 | 4,088 |  |
| **Vehicle type** |  |  |  |  |  |  |  |  |  |
| **Passenger car & van** | 3,124 | 24,647 | 5,022 | 4,651 | 2,265 | 2,464 | 35,645 | 5,960 | $\chi^{2}$=929.59  $p$<0.001 |
| **Pickup** | 543 | 2,951 | 607 | 514 | 267 | 193 | 3,681 | 794 |  |
| **Heavy vehicle** | 450 | 1,200 | 290 | 400 | 170 | 113 | 1,521 | 630 |  |

**Table A2 Chi-square tests of the D2 distribution (2013)**

| **Driver-vehicle groups** | **H1** | **H2** | **H3** | **H4** | **H5** | **H6** | **H7** | **H8** | **Chi-square test** |
| --- | --- | --- | --- | --- | --- | --- | --- | --- | --- |
| **Age** |  |  |  |  |  |  |  |  |  |
| **15-30** | 1,402 | 9,785 | 1,810 | 1,677 | 875 | 782 | 11,001 | 2,041 | $\chi^{2}$=653.49  $p$<0.001 |
| **31-60** | 3,444 | 15,503 | 3,201 | 3,271 | 1,533 | 1,635 | 25,366 | 4,413 |  |
| **>60** | 736 | 4,634 | 1,119 | 843 | 386 | 547 | 6,471 | 1,188 |  |
| **Gender** |  |  |  |  |  |  |  |  |  |
| **Female** | 2,197 | 14,410 | 2,888 | 2,630 | 1,308 | 1,589 | 21,592 | 3,273 | $\chi^{2}$=404.00  $p$<0.001 |
| **Male** | 3,385 | 15,512 | 3,242 | 3,161 | 1,486 | 1,375 | 21,246 | 4,369 |  |
| **Vehicle type** |  |  |  |  |  |  |  |  |  |
| **Passenger car & van** | 4,185 | 25,606 | 5,217 | 4,765 | 2,403 | 2,644 | 37,393 | 6,123 | $\chi^{2}$=1395.4  $p$<0.001 |
| **Pickup** | 705 | 2,998 | 648 | 506 | 249 | 213 | 3,792 | 825 |  |
| **Heavy vehicle** | 692 | 1,318 | 265 | 520 | 142 | 107 | 1,653 | 694 |  |
