# Supplementary material for: The difference in quasi-induced exposure to crashes involving various hazardous driving actions
Source: PLoS One. 2023 Feb 2;18(2):e0279387. doi: 10.1371/journal.pone.0279387 (PMC9894421; doi:10.1371/journal.pone.0279387)
Supplement: S1 File — (PDF) [file pone.0279387.s003.pdf]

CRASH REPORT INFORMATION SYSTEM

TRANSPORTATION CRASH MASTER

(285-Character Record)

Data File Description

Michigan Department of Transportation
Traffic and Safety Division
Technical Services Unit

P O Box 30050
Lansing, Michigan 48909

Revised January 11, 2002

CRASH REPORT INFORMATION SYSTEM

TRANSPORTATION CRASH MASTER

Abstract

The Transportation Crash Master is an extract of MDOT's CRIS (Crash Report Information System) data base. It is generated by the CRIS software system based on selection criteria and contains general information about each selected crash and up to the first three traffic units involved in the crash. It does not contain all the information available for those crashes; specifically it has no information that could be used to identify any individual involved in the crash.

Availability

This file is available to authorized traffic crash analysts and researchers who have an identified business need for the information. The file can be generated in several formats, including an ASCII flat file, a generic spreadsheet, or a generic data base.

This file can be generated only by certain authorized staff members of the Traffic and Safety Division.

The official keeper of traffic crash records is the Criminal Justice Information Center, Michigan State Police.

Note on Use

The user is responsible for knowing the selection criteria used to generate the file and for using and reporting the data properly. There is nothing within the file itself that identifies the selection criteria.

RESTRICTED USE WARNING
23 USC 409 AND 402(k)(1)

You are being provided information of which disclosure is restricted by federal law. Your access is conditioned upon your agreement to comply with the requirements of federal law. MDOT provides access to this information with the understanding that it will be used strictly for scientific research purposes and/or for governmental purposes by governmental units. MDOT authorizes no other use of this privileged information. MDOT does not waive any privilege based on this limited release of information.

It is the intent of these federal laws that this information not be disclosed, discovered or admitted into evidence for use in law suits for damages at locations addressed by this information. Privileged information includes information compiled or collected for the purpose of identifying, evaluating or planning safety enhancement projects and construction projects in addition to information contained in computerized safety record keeping systems which correlate traffic crash data with highway features.

Federal law at 23 USC 409 provides:

> Reports, surveys, schedules, lists, or data compiled or collected for the purpose of identifying, evaluating, or planning the safety enhancement of potential accident sites, hazardous roadway conditions, or railway-highway crossings ... or for the purpose of developing any highway safety construction improvement project which may be implemented utilizing Federal-aid highway funds shall not be subject to discovery or admitted into evidence in a Federal or State court proceeding or considered for other purposes in any action for damages arising from any occurrence at a location mentioned or addressed....

Federal law at 23 USC 402(k)(1) provides:

> Notwithstanding any other provision of law, if a report, list, schedule, or survey is prepared by or for a State of political subdivision thereof under this subsection ["a comprehensive computerized safety record keeping system designed to correlate data regarding traffic accidents, drivers, motor vehicles, and roadways"]. Such report, list, schedule, or survey shall not be admitted as evidence or used in any suit or action for damages arising out of any matter mentioned in such report, list, schedule, or survey.

Position	Format	Description
1	I1<br>[9]	**Region**<br>1 First Region - Superior<br>2 Second Region - North<br>3 Third Region - Grand<br>4 Fourth Region - Bay<br>5 Fifth Region - Southwest<br>6 Sixth Region - University<br>7 Seventh Region - Metro
2	I1<br>[9]	**District**<br>1 First District - Crystal Falls<br>2 Second District - Newberry<br>3 Third District - Cadillac<br>4 Fourth District - Alpena<br>5 Fifth District - Grand Rapids<br>6 Sixth District - Saginaw<br>7 Seventh District - Kalamazoo<br>8 Eighth District - Jackson<br>9 Ninth District - Southfield (Metro)
3 - 9	I7<br>[9999999]	**Physical Road Number**<br>A unique seven-digit code that identifies the road on which the crash occurred. No coding system is used for PR numbers.
10 - 14	F5.3<br>[99v999]	**Physical Road Milepoint**<br>Mileage along the physical road number, to 0.001 mile (last digit is usually zero). Implied decimal point between 2nd and 3rd digits.
15 - 19	I5<br>[99999]	**Control Section**<br>A five-digit code that identifies the portion of the trunkline system:<br>Digits 1 and 2 : MDOT County Number<br>Digits 3 thru 5 : The unique Trunkline segment number<br><br>If a crash occurs at the junction of two or more control sections, the control section of the higher route classification (I, US, M, I Business Loop or Spur, US Business Route, M Business Route) is used.<br><br>If the route classifications are the same, the lower numbered route is used.<br><br>If a crash occurs at a grade separation, it is coded to the trunkline that the vehicles are on.<br><br>If a crash occurs on a ramp or loop, it is coded to the major road control section.
20 - 24	F5.3<br>[99v999]	**Control Section Milepoint**<br>Mileage along the control section, to 0.001 mile (last digit is usually zero). Implied decimal point between 2nd and 3rd digits.

Position	Format	Description	
25	I1	**On Trunkline Indicator**	
	[9]	0	Unlocated & No
		1	Yes
26	I1	**Highway Area Type**	
	[9]	1	Interchange area (within ramp limits in all directions).
		2	Intersection area (normally within 150 feet in any direction from the intersection, but farther if the crash is attributable to the intersection).
		3	Non-intersection and non-interchange area.
		4	Non-traffic motor vehicle crash.
		NOTE: Intersection area also includes channelized intersections.	
27 - 28	I2	**Highway Area Code**	
	[99]		**On Freeway**
		00	Uncoded & Errors
		01	Entrance or Exit Ramp Related
		02	Median Crossing Related
		03	Transition Area
		04	Rest Area Related
		05	Scale or Weigh Station Related
		06	All Other Freeway Areas
			**Related to or Within 150' of an Intersection**
		07	Within Intersection
		08	Driveway Related Within 150' of Intersection
		09	Other Intersection Related
			**Other Area**
		10	Straight, Unrelated Others
		11	Curved Road, Unrelated Other
		12	Driveway Related
		13	Parking Related
		14	Transition Area
		15	Median Crossing Related
		16	Railroad Crossing Related
		17	Rest Area
		18	Scale or Weigh Station Related
		19	Non-Traffic Area
		20	Other Non-Intersection Area
		21	Unknown

CRASH REPORT INFORMATION SYSTEM
TRANSPORTATION CRASH MASTER
Data File Record Description

Position	Format	Description	
29	I1	**Relationship to Road**	
	[9]	0	Uncoded & Errors
		1	On the Road
		2	In the Median
		3	On the Shoulder
		4	Outside of the Shoulder or Curb
		5	In the Gore (Area between the ramp and freeway convergence)
		6	Unknown
30	I1	**Non-Traffic Crash Indicator**	
	[9]	0	No
		1	Yes
31	I1	**Route Class**	
	[9]	0	Not Located
		1	Interstate Route
		2	US Route
		3	M Route
		4	Interstate Business Loop or Spur
		5	US Business Route
		6	M Business Route
		7	Connector
		8	Service Drive
		9	County Road or City Street or Not Known
32 - 34	I3	**Route Number**	
	[999]	Actual MDOT trunkline number, preceded by leading zeros.	
		999	Local trafficway or type not known
35 - 36	I2	**Speed Limit**	
	[99]	99	Uncoded & Errors
37	I1	**Speed Limit Posted**	
	[9]	0	Uncoded & Errors
		1	Yes
		2	No

Position	Format	Description			
38 - 39	I2	**MDOT County Number**			
	[99]	01	Alcona	44	Lapeer
		02	Alger	45	Leelanau
		03	Allegan	46	Lenawee
		04	Alpena	47	Livingston
		05	Antrim	48	Luce
		06	Arenac	49	Mackinac
		07	Baraga	50	Macomb
		08	Barry	51	Manistee
		09	Bay	52	Marquette
		10	Benzie	53	Mason
		11	Berrien	54	Mecosta
		12	Branch	55	Menominee
		13	Calhoun	56	Midland
		14	Cass	57	Missaukee
		15	Charlevoix	58	Monroe
		16	Cheboygan	59	Montcalm
		17	Chippewa	60	Montmorency
		18	Clare	61	Muskegon
		19	Clinton	62	Newaygo
		20	Crawford	63	Oakland
		21	Delta	64	Oceana
		22	Dickinson	65	Ogemaw
		23	Eaton	66	Ontonagon
		24	Emmet	67	Osceola
		25	Genesee	68	Oscoda
		26	Gladwin	69	Otsego
		27	Gogebic	70	Ottawa
		28	Grand Traverse	71	Presque Isle
		29	Gratiot	72	Roscommon
		30	Hillsdale	73	Saginaw
		31	Houghton	74	Sanilac
		32	Huron	75	Schoolcraft
		33	Ingham	76	Shiawassee
		34	Ionia	77	St. Clair
		35	Iosco	78	St. Joseph
		36	Iron	79	Tuscola
		37	Isabella	80	Van Buren
		38	Jackson	81	Washtenaw
		39	Kalamazoo	82	Wayne
		40	Kalkaska	83	Wexford
		41	Kent	84	Uncoded & Errors
		42	Keweenaw		
		43	Lake		

Position	Format	Description			
40 - 41	I2	**City or Township**			
	[99]	A two-digit code identifying the local government agency.			
		00	Uncoded & Errors		
		Value: 01 thru 99			
		NOTE: If a city boundary extends over a county line, the city has the same City or Township code in both counties.			
42 - 45	I4	**Year of Crash**			
	[9999]	Four digits of the calendar year			
		Format: CCYY			
46 - 47	I2	**Month of Crash**			
	[99]	00	Uncoded & Errors	07	July
		01	January	08	August
		02	February	09	September
		03	March	10	October
		04	April	11	November
		05	May	12	December
		06	June	13	Uncoded & Errors
48 - 49	I2	**Day of Month**			
	[99]	00	Uncoded & Errors		
		Value: 01 thru 31			
		32	Uncoded & Errors		
50 - 51	I2	**Hour of Occurrence**			
	[99]	00	Midnight - 1am		
		01	1am - 2am	14	2pm - 3pm
		02	2am - 3am	15	3pm - 4pm
		03	3am - 4am	16	4pm - 5pm
		04	4am - 5am	17	5pm - 6pm
		05	5am - 6am	18	6pm - 7pm
		06	6am - 7am	19	7pm - 8pm
		07	7am - 8am	20	8pm - 9pm
		08	8am - 9am	21	9pm - 10pm
		09	9am - 10am	22	10pm - 11pm
		10	10am - 11am	23	11pm - Midnight
		11	11am - Noon	24	Midnight
		12	Noon - 1pm	77	Uncoded & Errors
		13	1pm - 2pm	99	Unknown
52	I1	**Weekday**			
	[9]	0	Error in Date	4	Wednesday
		1	Sunday	5	Thursday
		2	Monday	6	Friday
		3	Tuesday	7	Saturday

Position	Format	Description	
53	I1	**Number of Motor Vehicles**	
	[9]	Value: 1 thru 9	
54 - 55	I2	**MDOT Crash Type**	
	[99]	00	Uncoded & Errors
		11	Overturn
		12	Hit Train
		13	Pedestrian
		14	Bicycle
		15	Fixed Object
		16	Other Object
		17	Hit Parked Vehicle
		18	Animal
		19	Miscellaneous Single Vehicle
		20	Miscellaneous Multiple Vehicle
		21	Angle Straight
		22	Angle Turn
		23	Head On Left Turn
		24	Rear End Straight
		25	Rear End Left Turn
		26	Rear End Right Turn
		27	Dual Left Turn
		28	Dual Right Turn
		31	Head On
		32	Side-Swipe Same
		33	Side-Swipe Opposite
		34	Angle Drive
		35	Rear End Drive
		36	Other Drive
		37	Backing
		38	Parking
56	I1	**Crash Injury Severity**	
	[9]	0	Uncoded & Errors
		1	Fatal
		2	Injury
		3	Property Damage Only
57	I1	**Crash Greatest Injury Severity**	
	[9]	0	Uncoded & Errors
		1	Killed
		2	Incapacitating Injury
		3	Non-Incapacitating Injury
		4	Possible Injury
		5	No Injury

Position	Format	Description
58 - 59	I2	**Number of Persons Killed in Crash**
	[99]	Value: 00 thru 98
		99 Uncoded & Errors
60 - 61	I2	**Number of A [Incapacitating] Injuries in Crash**
	[99]	Value: 00 thru 98
		99 Uncoded & Errors
62 - 63	I2	**Number of B [Non-Incapacitating] Injuries in Crash**
	[99]	Value: 00 thru 98
		99 Uncoded & Errors
64 - 65	I2	**Number of C [Possible] Injuries in Crash**
	[99]	Value: 00 thru 98
		99 Uncoded & Errors
66 - 67	I2	**Number of Persons Injured in Crash**
	[99]	Value: 00 thru 98
		99 Uncoded & Errors
68 - 69	I2	**Number of Persons Un-Injured in Crash**
	[99]	Value: 00 thru 98
		99 Uncoded & Errors
70 - 71	I2	**Number of Occupants in Crash**
	[99]	Value: 01 thru 98
		99 Uncoded & Errors
72	I1	**Weather Condition**
	[9]	0 Uncoded & Errors
		1 Clear
		2 Cloudy
		3 Fog or Smoke
		4 Rain
		5 Snow or Blowing Snow
		6 Severe Wind
		7 Sleet or Hail
		8 Other
73	I1	**Lighting**
	[9]	0 Uncoded & Errors
		1 Daylight
		2 Dawn
		3 Dusk
		4 Dark, Lighted
		5 Dark, Unlighted
		6 Other

Position	Format	Description	
74	I1	**Roadway Surface Condition**	
	[9]	0	Uncoded & Errors
		1	Dry
		2	Wet
		3	Icy
		4	Snowy
		5	Muddy
		6	Slushy
		7	Debris
		8	Other
75	I1	**Traffic Control**	
	[9]	0	Uncoded & Errors
		1	Signal
		2	Stop Sign
		3	Yield Sign
		4	None
76	I1	**Work Zone Type**	
	[9]	0	Uncoded & Errors
		1	Construction or Maintenance
		2	Utility
77	I1	**Work Zone Lane Closure**	
	[9]	0	Uncoded & Errors
		1	Yes
		2	No
78	I1	**Work Zone Activity**	
	[9]	0	Uncoded & Errors
		1	On Road
		2	Off Road
		3	None
79	I1	**Drinking Involved in Crash**	
	[9]	0	No
		1	Yes
80	I1	**Truck or Bus Involved in Crash**	
	[9]	0	No
		1	Yes
81	I1	**Snowmobile Involved in Crash**	
	[9]	0	No
		1	Yes

Position	Format	Description	
82	I1	**Emergency Vehicle Involved in Crash**	
	[9]	0	No
		1	Yes
83	I1	**ORV Involved in Crash**	
	[9]	0	No
		1	Yes
84	I1	**Pedestrian Involved in Crash**	
	[9]	0	No
		1	Yes
85	I1	**Bicyclist Involved in Crash**	
	[9]	0	No
		1	Yes
86	I1	**Farm Equipment Involved in Crash**	
	[9]	0	No
		1	Yes
87	I1	**Deer Involved in Crash**	
	[9]	0	No
		1	Yes
88	I1	**School Bus Involved in Crash**	
	[9]	0	No
		1	Yes
89	I1	**Motorcycle Involved in Crash**	
	[9]	0	No
		1	Yes
90	I1	**Train Involved in Crash**	
	[9]	0	No
		1	Yes
91	I1	**Hit and Run Crash**	
	[9]	0	No
		1	Yes
92	I1	**Fleeing Situation Crash**	
	[9]	0	No
		1	Yes
93	I1	**Public Property Damaged**	
	[9]	0	Uncoded & Errors
		1	Yes
		2	No

Position	Format	Description

> NOTE: This file contains information of the first three traffic units involved in the crash, described in the next 21 data fields (34 characters per traffic unit):
> 94 - 127 Data for Traffic Unit # 1
> 128 - 161 Data for Traffic Unit # 2
> 162 - 195 Data for Traffic Unit # 3
> If there is no traffic unit #2 or #3, the fields for that traffic unit are blank.

Position	Format	Description	
94 - 95	I2	**Type, Unit 1**	
128 - 129	[99]	**Type, Unit 2**	
162 - 163		**Type, Unit 3**	
		00	Uncoded & Errors
		01	Passenger Car and Station Wagon
		02	Van or Motorhome
		03	Pickup
		04	Truck under 10,000 lbs.
		05	Cycle
		06	Moped
		07	Go-Cart
		08	Snowmobile
		09	ORV or ATV
		10	Other Non-Commercial
		11	Miscellaneous Commercial
		12	AA: Combo Unit
		13	AH: Combo, Hazardous
		14	AN: Combo, Tank
		15	AP: Combo, Passenger
		16	AT: Combo, Double or Triple
		17	AX: Combo, Tank and Hazardous
		18	AY: Combo, Double or Triple Tank
		19	AZ: Combo, Double or Triple Hazardous
		20	AL: Combo, Double or Triple Hazardous Tank
		21	BB: Single over 26k
		22	BH: Single, Hazardous
		23	BN: Single, Tank
		24	BP: Single, Passenger
		25	BX: Single, Tank and Hazardous
		26	CH: Under 26k, Hazardous
		27	CP: Under 26k, Passenger
		28	CX: Under 26k, Tank and Hazardous
		29	Other Commercial

Position	Format	Description	
96	I1	**Special Vehicle Category, Unit 1**	
130	[9]	**Special Vehicle Category, Unit 2**	
164		**Special Vehicle Category, Unit 3**	
		0	Uncoded & Errors
		1	Police
		2	Fire
		3	Bus
		4	Ambulance
		5	Farm
		6	Construction
97 - 98	I2	**Use, Unit 1**	
131 - 132	[99]	**Use, Unit 2**	
165 - 166		**Use, Unit 3**	
		00	Uncoded & Errors
		01	Private
		02	Commercial
		03	In Pursuit or Emergency (in use)
		04	Farm
		05	School or Education
		06	Club or Church ("Y" Tag)
		07	Military Vehicle
		08	Other Government, Non-Emergency
		09	Utility
		10	Road Construction or Maintenance
		11	Other
99	I1	**Non-Commercial Trailer, Unit 1**	
133	[9]	**Non-Commercial Trailer, Unit 2**	
167		**Non-Commercial Trailer, Unit 3**	
		0	Uncoded & Errors
		1	Utility
		2	House
		3	Boat
		4	Farm
		5	Towed Auto
		6	Recreational Double
		7	Other

Position	Format	Description			
100	I1	**Defect, Unit 1**			
134	[9]	**Defect, Unit 2**			
168		**Defect, Unit 3**			
		0	Uncoded & Errors		
		1	Brakes		
		2	Lights or Reflectors		
		3	Steering		
		4	Tires or Wheels		
		5	Windows		
		6	Other		
101 - 102	I2	**Worst Impact, Unit 1**			
135 - 136	[99]	**Worst Impact, Unit 2**			
169 - 170		**Worst Impact, Unit 3**			
		00	Rollover	07	Driver Side
		01	Front Center	08	Front Left Corner
		02	Front Right Corner	09	Undercarriage
		03	Passenger Side	10	Multiple Areas
		04	Right Rear Corner	11	Other or Unknown
		05	Rear Center	12	None
		06	Rear Left Corner	99	Uncoded & Errors
103	I1	**Unit 1 Driveable**			
137	[9]	**Unit 2 Driveable**			
171		**Unit 3 Driveable**			
		0	Uncoded & Errors		
		1	Yes		
		2	No		
104 - 106	I3	**Age of Driver 1** (or pedestrian, bicyclist, etc.)			
138 - 140	[999]	**Age of Driver 2**			
172 - 174		**Age of Driver 3**			
		999	Unknown		
107	A1	**Sex of Driver 1** (or pedestrian, bicyclist, etc.)			
141	[X]	**Sex of Driver 2**			
175		**Sex of Driver 3**			
		‘Space’	Uncoded & Errors		
		M	Male		
		F	Female		
108	I1	**Drinking, Driver 1** (or pedestrian, bicyclist, etc.)			
142	[9]	**Drinking, Driver 2**			
176		**Drinking, Driver 3**			
		0	Uncoded & Errors		
		1	Yes		
		2	No		

CRASH REPORT INFORMATION SYSTEM
TRANSPORTATION CRASH MASTER
Data File Record Description

Position	Format	Description	
109 - 110	I2	**Condition, Driver 1** (or pedestrian, bicyclist, etc.) (1999)	
143 -144	[99]	**Condition, Driver 2**	
177 - 178		**Condition, Driver 3**	
		(Reserved for Future Use)	
		00	Uncoded & Errors
		01	Appeared Normal
		02	Had Been Drinking
		03	Illegal Drug Use
		04	Sick
		05	Fatigue
		06	Asleep
		07	Medication
		08	Driver Distracted
		09	Driver Using Cellular Phone
		99	Unknown
111	I1	**Degree of Injury to Driver 1** (or pedestrian, bicyclist, etc.)	
145	[9]	**Degree of Injury to Driver 2**	
179		**Degree of Injury to Driver 3**	
		0	Uncoded & Errors
		1	Fatal
		2	Type A (Incapacitating) Injury
		3	Type B (Non-Incapacitating) Injury
		4	Type C (Possible) Injury
		5	No Injury
112 - 113	A2	**Direction of Travel** (before crash)**, Unit 1**	
146 - 147	[XX]	**Direction of Travel** (before crash)**, Unit 2**	
180 - 181		**Direction of Travel** (before crash)**, Unit 3**	
		‘Space’	Uncoded & Errors
		N	North
		NW	Northwest
		SE	Southeast
		E	East
		NE	Northeast
		S	South
		SW	Southwest
		W	West

Position	Format	Description	
114 - 115	I2	**Action Prior to Crash, Driver 1** (or pedestrian, bicyclist, etc.)	
148 - 149	[99]	**Action Prior to Crash, Driver 2**	
182 - 183		**Action Prior to Crash, Driver 3**	
		00	Uncoded & Errors
		01	Going Straight
		02	Turning Left
		03	Turning Right
		04	Stopped on Road
		05	In Prior Crash
		06	Changing Lanes
		07	Backing
		08	Slowing or Stopped on Road
		09	Slowing or Stopped Other
		10	Starting Up on Road
		11	Starting Up Other
		12	Entering Parking
		13	Leaving Parking
		14	Entering Road
		15	Leaving Road
		16	Making U-Turn
		17	Overtaking or Passing
		18	Avoiding Object
		19	Avoiding Pedestrian
		20	Avoiding Vehicle (Front or Back)
		21	Avoiding Vehicle (Angle)
		22	Driverless Moving
		23	Parked
		24	Crossing at Intersection
		25	Crossing Midblock
		26	Getting On or Off Vehicle
		27	In Road With Traffic
		28	In Road Against Traffic
		29	Standing or Laying in Road
		30	Pushing or Working on Vehicle
		31	Other Work in Road
		32	Playing in Road
		33	Other Reason in Road
		34	Not in Road
		35	Other
		36	Unknown
		37	Avoiding Animal (1999)

Position	Format	Description	
116 - 117	I2	**Harmful Event #1, Unit 1**	
150 - 151	[99]	**Harmful Event #1, Unit 2**	
184 - 185		**Harmful Event #1, Unit 3**	
			**Non Collision**
		00	Uncoded & Errors
		01	Loss of Control
		02	Crossed Centerline or Median
		03	Ran Off Road Left
		04	Ran Off Road Right
		05	Re-Entered Road
		06	Overturn
		07	Separation of Units
		08	Fire or Explosion
		09	Immersion
		10	Jackknife
		11	Downhill Runaway
		12	Cargo Loss or Shift
		13	Individual Fell Off
		14	Other Non-Collision
			**Collision With Non Fixed**
		15	Hit Pedestrian
		16	Hit Pedalcycle
		17	Hit Motor Vehicle in Transport
		18	Hit Parked Vehicle
		19	Hit Railroad Train
		20	Hit Animal
		21	Hit Other Non-Fixed Object

Position	Format	Description	
			**Collision With Fixed**
		22	Hit Bridge, Pier or Abutment
		23	Hit Bridge Parapet End
		24	Hit Bridge Rail
		25	Hit Guardrail Face
		26	Hit Guardrail End
		27	Hit Median Barrier
		28	Hit Traffic Sign Post
		29	Hit Traffic Signal Post
		30	Hit Luminaire Support
		31	Hit Utility Pole
		32	Hit Other Pole
		33	Hit Culvert
		34	Hit Curb
		35	Hit Ditch
		36	Hit Embankment
		37	Hit Fence
		38	Hit Mailbox
		39	Hit Tree
		40	Hit Railroad Crossing Signal
		41	Hit Building
		42	Hit Traffic Island
		43	Hit Fire Hydrant
		44	Hit Impact Attenuator
		45	Hit Other Fixed Object
118 - 119	I2	**Harmful Event #2, Unit 1**	
152 - 153	[99]	**Harmful Event #2, Unit 2**	
186 - 187		**Harmful Event #2, Unit 3**	
		(Coding is the same as Harmful Event #1)	
120 - 121	I2	**Harmful Event #3, Unit 1**	
154 - 155	[99]	**Harmful Event #3, Unit 2**	
188 - 189		**Harmful Event #3, Unit 3**	
		(Coding is the same as Harmful Event #1)	
122 - 123	I2	**Harmful Event #4, Unit 1**	
156 - 157	[99]	**Harmful Event #4, Unit 2**	
190 - 191		**Harmful Event #4, Unit 3**	
		(Coding is the same as Harmful Event #1)	
124	I1	**Event Most Harmful, Unit 1**	
158	[9]	**Event Most Harmful, Unit 2**	
192		**Event Most Harmful, Unit 3**	
		0	Uncoded & Errors
		Value: 1 thru 4	

Position	Format	Description	
125 - 126	I2	**Hazardous Action, Driver 1** (or pedestrian, bicyclist, etc.)	
159 - 160	[99]	**Hazardous Action, Driver 2**	
193 - 194		**Hazardous Action, Driver 3**	
		00	None
		01	Speed Too Fast
		02	Speed Too Slow
		03	Failed to Yield
		04	Disobeyed Traffic Control
		05	Drove Wrong Way
		06	Drove Left of Center
		07	Improper Passing
		08	Improper Lane Use
		09	Improper Turn
		10	Improper Signal
		11	Improper Backing
		12	Fail to Stop in Assured Clear Distance
		13	Other
		14	Unknown
		15	Reckless Driving (1999)
		16	Careless or Negligent Driving (1999)
		99	Uncoded & Errors
127	I1	**Violator, Driver 1** (or pedestrian, bicyclist, etc.)	
161	[9]	**Violator, Driver 2**	
195		**Violator, Driver 3**	
		0	No
		1	Yes
196 - 225	A30	**Road Name On**	
	[X(30)]	English Name of the road or street that crash occurred on, as recorded by the investigating officer.	
226 - 230	I5	**Distance From Crossroad**	
	[99999]	Reported distance from crash location to reference point in feet, as recorded by the investigating officer.	

Position	Format	Description
231 - 232	A2<br>[XX]	**Direction from Crossroad**<br>'Space' Uncoded & Errors<br>BR Begin Ramp<br>E East<br>ER End Ramp<br>N North<br>NE Northeast<br>NW Northwest<br>S South<br>SE Southeast<br>SW Southwest<br>X At Intersection<br>W West<br><br>Reported direction from reference point to crash location, as recorded by the investigating officer.<br><br>'X' Crash occurred exactly at the reference point.<br><br>NOTE: The reference point is the point where the center lines of the roads meet. Crashes occurring within a paved intersection will be referenced by distance and direction from that point (such as '20 ft SE'). Few if any crashes occur at the intersection reference point.
233 - 262	A30<br>[X(30)]	**Intersecting Crossroad**<br>English Name of the reference point, as recorded by the investigating officer.
263 - 269	A7<br>[X(7)]	**Crash Report Number**<br>Unique serial number for the crash. Changed to alpha numeric as of 1998.
270 - 272	A3<br>[X(3)]	**Microfilm Reel Number**<br>Reel Number where microfilm copy of original UD-10 crash report is stored.
273 - 276	A4<br>[X(4)]	**Microfilm Frame Number**<br>Frame Number where microfilm copy of original UD-10 crash report is stored.
277 - 285	A9<br>[X(9)]	**Policing Agency ORI Number**<br>First two characters denote the Policing Agency State, "MI"<br>Characters three and four denote the Policing Agency State Police County Number<br>Characters five through nine denote the Policing Agency Number

– End of Record –
